# Supplementary material for: Quantitative Analysis of Isopimpinellin from Ammi majus L. Fruits and Evaluation of Its Biological Effect on Selected Human Tumor Cells
Source: Molecules. 2024 Jun 17;29(12):2874. doi: 10.3390/molecules29122874 (PMC11206288; doi:10.3390/molecules29122874)
Supplement: Supplementary file 1 [file molecules-29-02874-s001.zip › molecules-3022047-supplementary.pdf]

Quantitative Analysis of Isopimpinellin from *Ammi majus* L. Fruits and Evaluation of Its Biological Effect on Selected Human Tumor Cells

Magdalena Bartnik [1,*], Adrianna Sławińska-Brych [2], Magdalena Mizerska-Kowalska [3], Anna Karolina Kania [1] and Barbara Zdzisińska [3]

1 Department of Pharmacognosy with Medicinal Plants Garden, Medical University of Lublin,
Chodźki 1 Street, 20-093 Lublin, Poland; amazurek02@interia.pl (A.K.K.)
2 Department of Cell Biology, Institute of Biological Sciences, Maria Curie-Skłodowska University,
Akademicka 19 Street, 20-033 Lublin, Poland; adrianna.slawinska-brych@mail.umcs.pl
3 Department of Virology and Immunology, Institute of Biological Sciences, Maria Curie-Skłodowska
University, Akademicka 19 Street, 20-033 Lublin, Poland; magdalena.mizerska-kowalska@mail.umcs.pl (M.M.-K.); barbara.zdzisinska@mail.umcs.pl (B.Z.)
* Correspondence: magdalenabartnik@umlub.pl

Table S1. Summary of data on cell lines used in experiments

**Cell line**	**HOS**	**Saos-2**	**HT-29**	**SW620**	**RPMI8226**	**U266**
**Phenotypic characteristics**						
**cc character**						
**Tumour source**	primary	primary	primary	metastatic - lymph node	primary	primary
**Histology**	osteosarcoma	osteosarcoma	colon carcinoma	colon adenocarcinoma	plasmacytoma myeloma	plasmacytoma myeloma
**Morphology**	epithelial-like	epithelial-like	epithelial	epithelial	lymphoblastic	lymphoblastic
**Growth mode**	adherent	adherent	adherent	adherent	non-adherent	non-adherent
**Migration/ invasiveness (in vivo)**	+++	++	++	+++	+	+
**Gene mutation**						
APC	-	-	+	+	-	-
BRAF	-	-	+	-	-	+
CDKN2A	+	-	-	-	-	-
EGFR	-	-	-	-	+	-
PIK3CA	-	-	+	-	-	-
KRAS	-	-	-	+	+	-
SMAD4	-	-	+	+	-	-
TP53	+	+	+	+	+	+
RB1	-	+	-	-	-	+

Abbreviations: APC (adenomatous polyposis coli); BRAF (V-Raf Murine Sarcoma Viral Oncogene Homolog B); TP53 (tumor protein p53); RB1 (retinoblastoma1/RB transcriptional corepressor 1); and CDKN2A (cyclin-dependent kinase inhibitor 2A)/tumor suppressor gene).

1. Lauvrak, S.U.; Munthe, E.; Kresse, S.H.; Stratford, E.W.; Namløs, H.M.; Meza-Zepeda, L.A.; Myklebost, O. Functional characterisation of osteosarcoma cell lines and identification of mRNAs and miRNAs associated with aggressive cancer phenotypes. *Br. J. Cancer.* **2013**, *109(8),* 2228-36. doi: 10.1038/bjc.2013.549.

2. Zhu, X.L.; Liang, L.; Ding, Y.Q. [Expression of FMNL2 and its relation to the metastatic potential of human colorectal cancer cells]. *Nan Fang Yi Ke Da Xue Xue Bao*. **2008**, *28(10),* 1775-1778. Chinese. PMID: 18971169.

3. Jiang, S.; Zhou, F.; Zhang, Y.; Zhou, W.; Zhu, L.; Zhang, M.; Luo, J.; Ma, R.; Xu, X.; Zhu, J.; Dong, X.; Zhang, S.; Fang, J.; Sun, J.; Yang, X. Identification of tumorigenicity-associated genes in osteosarcoma cell lines based on bioinformatic analysis and experimental validation. *J. Cancer.* **2020**, *11(12),* 3623-3633. doi: 10.7150/jca.37393.

4. https://cancer.sanger.ac.uk/cosmic

5. https://maayanlab.cloud/Harmonizome/dataset/COSMIC+Cell+Line+Gene+Mutation+Profiles
